# Opening up a tailored tutor qualification program for medical students to other healthcare students – a mixed-method study

**DOI:** 10.1186/s12909-022-03304-y

**Published:** 2022-04-06

**Authors:** A. Homberg, S. Ziegler, C. Mahler, J. H. Schultz, S. Loukanova, J. Hundertmark

**Affiliations:** 1 Department of Medical Education Research, Medical Faculty Mannheim at Heidelberg University, Theodor-Kutzer-Ufer 1-3, 68176 Mannheim, Germany; 2 Department of General Practice and Health Services Research, Heidelberg University Hospital, Im Neuenheimer Feld 130.3, 69120 Heidelberg, Germany; 3 Department of Nursing Science, Tübingen University Hospital, Hoppe-Seyler-Str. 9, 72076 Tübingen, Germany; 4 Department of General Internal Medicine and Psychosomatics, Heidelberg University Hospital, Im Neuenheimer Feld 410, 69120 Heidelberg, Germany

**Keywords:** Interprofessional education, Peer teaching, Peer-assisted learning, Tutor qualification, Undergraduate education

## Abstract

**Background:**

Peer-led tutorials are widely used in medical education to promote practical skills acquisition and support faculty staff. Typically, student tutors are custom trained for this specific task. We investigated whether opening up an existing medical tutor qualification program to other degree programs is successful in terms of acceptance among students, acquisition of tutor-specific and interprofessional competencies, and which factors contribute to success or failure.

**Methods:**

We developed a two-day tutor qualification program and conducted it annually from 2016 to 2020 with medical and other healthcare students. At the end of each course, we administered a written survey in which the participants rated the following items: their attitudes towards interprofessional learning (using the UWE-IP-D Interprofessional Learning Scale), the interprofessional learning setting, the teaching approach, and their competency acquisition (each on a five-point Likert scale; 1 = strongly agree, 5 = strongly disagree). Furthermore, we assessed participants’ qualitative feedback in free-text fields and performed inductive content analyses.

**Results:**

The study participation rate was high (response rate 97%; medical students: *n* = 75; healthcare students: *n* = 22). Participants stated high levels of competency acquisition (total *M* = 1.59, individual items’ *M’s* ranging from 1.20 to 2.05) and even higher satisfaction with the teaching approach (total *M* = 1.28, individual items’ *M’s* ranging from 1.43 to 1.05). Overall satisfaction with the training was *M* = 1.22; *SD* = 0.58. No significant differences in ratings were found between the student groups. The qualitative results showed that students appreciated the interprofessional setting and experienced it as enriching. The most positive feedback was found in didactics/teaching methods on role-plays and group work; most suggestions for improvement were found in the area of structure and organisation on breaks and time management.

**Conclusions:**

Opening up an existing medical tutor qualification program to other student groups can be seen as fruitful to teach not only tutor-related aspects but also interprofessional competencies. The results demonstrate the importance of detailed planning that considers group composition and contextual conditions and provides interactive teaching methods to promote interprofessional experiences. This study offers important information about prerequisites and methodological implementation that could be important for the interprofessional redesign of existing training programs.

**Supplementary Information:**

The online version contains supplementary material available at 10.1186/s12909-022-03304-y.

## Background

Student tutorial courses represent an essential element within undergraduate medical teaching today. One crucial and widespread setting is near-peer teaching. Experienced and trained students (referred to as tutors) teach small student groups in communication and clinical skills, for example [1–3]. Peer teaching has a long history in medical education [4, 5]. One major impetus for implementation was faculty dissatisfaction with large lectures where students played a passive role [6]. Peer teaching programs offer numerous further advantages to traditional faculty-led classes, for example, by addressing the faculty resource problem of providing multiple teachers to lead smaller group sizes for practising hands-on skills [7–11]. Peer-led courses can be as effective as traditional staff-led courses [12–14]. When students learn from students who usually have only a few semesters’ head start, a shared knowledge base and experience can be built upon [15]. Since tutors are generally highly motivated and engaged [16] and have high cognitive and social congruence [15], their familiarity with the learner’s current knowledge and learning situation can create a more personal and friendly learning atmosphere. This contributes to deepening and broadening the learners’ practical, cognitive and social skills and thus to quality assurance and professionalism [17]. To achieve these positive effects, both sensitive planning and implementation of tutorial courses and structured and rigorous training of tutors are necessary [18, 19]. A variety of strategies exist to prepare tutors for teaching other students.

Currently, medical education sees a trend towards a needs-adapted training strategy [20] as teaching in the medical program is characterised by a tightly paced curriculum and many competencies to be acquired. The high level of interactivity, examination of one’s role, and generally well-supported tutor training programs make this setting attractive for interprofessional education (IPE). Opening up proven and tailored medical teaching programs to students from other health professions can broaden participants’ horizons but risks weakening the focus on profession-specific competencies. In the field of tutorial courses, there is evidence that well-established monoprofessional approaches can be successfully transformed into interprofessional ones [21].

A fundamental, competency-based reorientation of medical studies in Germany is currently underway, calling for more robust integration of interprofessional teaching programs [22]. The aim is to ensure the quality of future cross-professional patient care [23, 24]. Being educated in an interprofessional setting provides an opportunity for professionals to share skills and knowledge that facilitates the development of shared values and a better understanding of the roles and responsibilities of the other healthcare students [25]. According to the definition given by the Centre for the Advancement of Interprofessional Education, “Interprofessional learning takes place when members or students of two or more professions learn with, from and about each other to improve collaboration and the quality of care and services” [26]. The collaboration for which IPE prepares is more than cooperation since students learn to empower each other in a nurturing and mutually supportive environment to collaborate flexibly and effectively across predetermined professional boundaries [27]. A form of leadership based on joint decision-making is required to ensure good cooperation within an interprofessional team, which poses a challenge to lecturers with traditional, hierarchical role models [28]. Hierarchical gradients between student tutors and student learners are at a minimum [29], and thus tutors and learners gradually build an interactional relationship [30]. Interprofessional peer teaching opens up the possibility of breaking down traditional role patterns. Interprofessional trained student tutors can serve as multipliers and role models for other students and are, therefore, of particular interest for spreading positive interprofessional attitudes. Opening up existing medical tutor qualification programs to other healthcare students could provide new interprofessional learning opportunities.

The following questions guide the study presented here:

- Do general attitudes differ toward interprofessional learning between tutors from different degree programs and toward other students?
- How is interprofessional tutor training perceived from the participating students’ point of view? Are there differences between different student groups? How high is participants overall satisfaction?
- Can the training program’s learning objectives be achieved in an interprofessional setting for all student groups, even though tutors are trained for different tutorials? Are there differences in goal achievement between the student groups?
- Which teaching methods and conditions contribute to the success or failure of interprofessional tutor training?

## Methods

### Peer teaching at the Heidelberg Medical Faculty

#### Medical students

Tutors are firmly integrated into the preclinical study phase at the Heidelberg Medical Faculty. They independently guide small groups of ten to twelve students in the training of medical skills, in particular, taking medical histories, physical examinations and venipuncture. Central coordination was established to successfully implement AaL^*Plus*^, the Living Anatomy plus Anamnesis program, to regulate the extensive coordination within the Medical Faculty and the evaluation of the acquired skills [31]. The program sustainably contributes to quality assurance, professionalism, and solving the problem of resources in medical education [32, 33]. As a prerequisite for teaching their younger peers, AaL^*Plus*^ tutors must complete basic training in didactics/teaching methods and group leadership, as well as additional doctor-patient communication and physical examination training [34]. These modules are part of a longitudinally structured program for tutor qualification, developed at the Heidelberg Medical Faculty based on a needs assessment and implemented in the 2010 summer semester [35]. The program consists of other advanced modules, such as subject-specific training, collegial advice, and reflection on the tutors’ activities. The basic tutor training is conducted annually before the winter semester. The Department of General Practice & Health Services Research at Heidelberg University’s Faculty of Medicine organises the AaL^*Plus*^ program. It conducts the corresponding tutor qualification program each year before the start of the winter semester.

#### Healthcare students

Two study programs for healthcare students are also affiliated with this department. The undergraduate bachelor’s degree program in “Interprofessional Health Care” was introduced in 2011. In eight semesters, students can complete an academic degree parallel to vocational training, such as nursing, physiotherapy or speech therapy [36]. In addition, the two-year, full-time Master of Science program in “Health Services Research and Implementation Science” was introduced in 2015. This program is unique because it focuses specifically on healthcare delivery in all settings, e.g., hospitals, primary care, ambulatory practices, and public health organisations [37]. Healthcare students (HCS) acquire basic knowledge of qualitative and quantitative research in both degree programs, but have very different learning backgrounds within the courses. It proved necessary to provide them with supervised sessions to practice scientific skills such as seeking literature and scientific writing. Individual modules, such as Medical English [38, 39], are also conducted interprofessionally, with integrated tutoring.

#### Developing the interprofessional tutor qualification program

Since the cohorts in both HCS degree programs are relatively small compared to the medical school and no direct experience with the training of tutors was available, the idea of joint tutor training within the Medical Faculty emerged. Moreover, one further aim was to establish additional opportunities for interprofessional learning to address the increasing need for interprofessional collaboration among healthcare professionals [40]. The respective coordinators of the healthcare degree programs and the established medical tutor qualification program first discussed the potential for a joint tutor qualification program in 2015. The medical tutor qualification program coordinators expressed concerns that the study groups could be too diverse and that the tailored tutoring qualification of medical students could be endangered due to the different group compositions and tutoring settings they were to be qualified for. In general, developing a training program for interprofessional student tutors requires excellent care so that students can later conduct tutorials on an equal footing and are accepted equally by all participants [41]. At the same time, all coordinators saw the opportunity to use existing resources and were willing to create interprofessional learning opportunities. Since tutors are role models, positive attitudes towards interprofessional learning and working can positively impact other students. In addition, the acquisition of interprofessional competencies in an interprofessional tutor qualification program makes it easier to establish other interprofessional peer-assisted teaching programs, for example, in communication training or scientific writing. An interprofessional tutor qualification program was developed with internal funding from the Heidelberg Medical Faculty, supported by the State Ministry of Baden-Württemberg for Science, Research and the Arts (“Sonderlinie Medizin” project). Tight curricula and, therefore time constraints necessitated a closely coordinated team of representatives from the participating programs working together to condense relevant content into a joint, comprehensive training program.

**Fig. 1:**
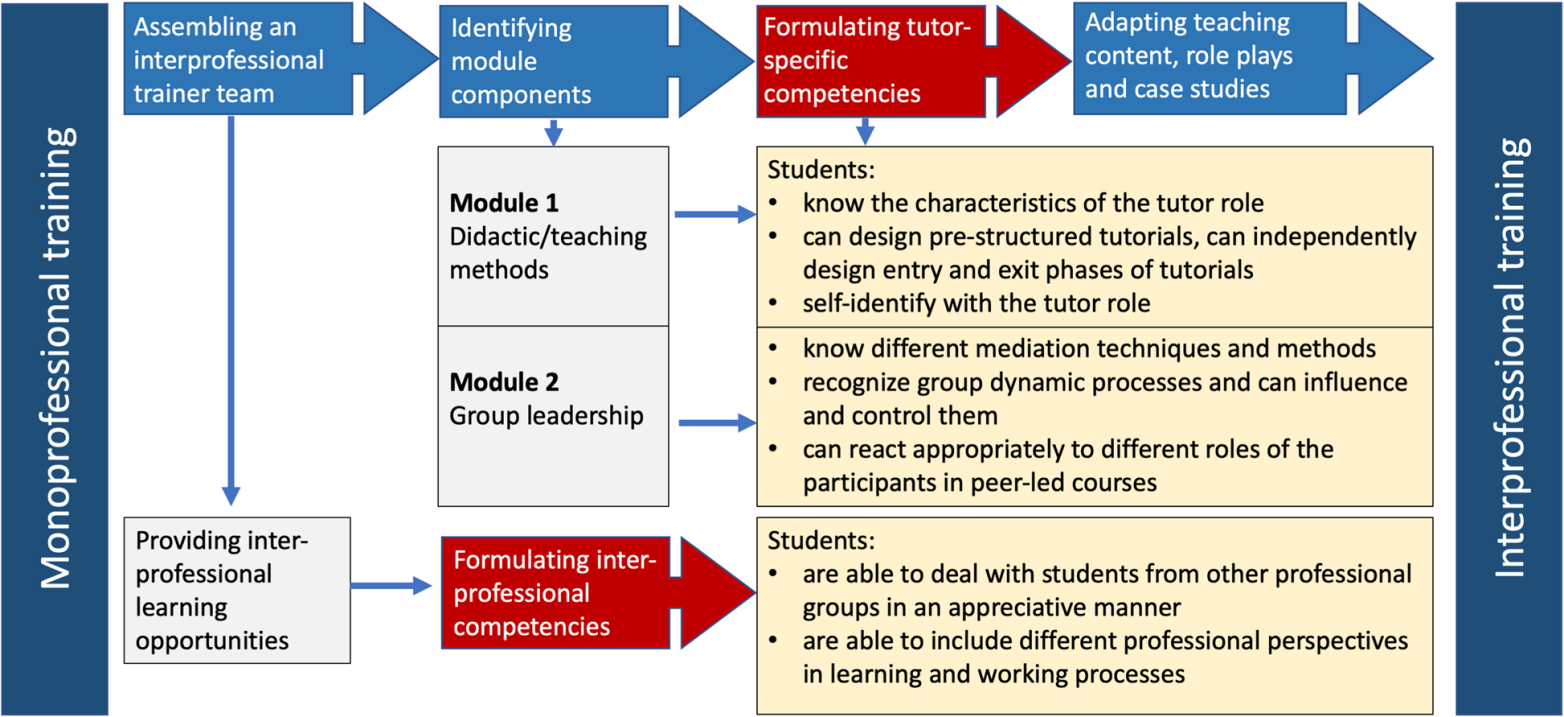
Development process and competencies of the interprofessional tutor qualification program

First, the multiprofessional trainer team, consisting of a medical educator, a psychologist, a physician, and a social anthropologist, assessed the essential training to identify the module components relevant to all degree programs. The trainers formulated competencies that all participants should acquire (Fig. 1).

In the next step the trainers reviewed all teaching content to ensure that it was understandable and authentic for the participating students. They transformed role plays and case studies into interprofessional scenarios and avoided profession-specific technical language. After a year-long planning phase, the team conducted a one-day module and evaluated it at the beginning of the 2015 winter semester. Evaluation results showed a high acceptance among the participating students. Based on the evaluation results, the team developed a two-day interprofessional tutor qualification program conducted annually from 2016 onwards. The four trainers prepared and conducted all training sessions, bringing to bear their professional perspectives. Table 1 shows the schedule of the newly designed interprofessional tutor qualification program. Training content and methods were continuously adapted in subsequent years, although the objectives and basic structure have been largely retained.

**Table 1:** Schedule of the interprofessional tutor qualification program

Schedule	Components	Teaching methods
First day: Didactics/teaching methods		
40 min	Getting to know and learn about each other	Sociometric formation, speed dating
20 min	Expectations	Table group and plenum session
60 min	Tasks and roles of a tutor	Table group and plenum session
30 min	Basics of learning, teaching and didactics [42–45]	Lectures
90 min	Design of entry and exit phases of tutorials	Role play
60 min	Motivation of participants, experiences of current tutors	Plenum discussion with experienced tutors
Second day: Group leadership		
60 min	Previous experience with groups and leadership	Circle of chairs, exchange, reflection
30 min	Perception exercises	Moving exercise
60 min	Rank dynamics, team roles and group phases [46, 47]	Theoretical input and role play
30 min	Group leadership and leadership styles [48]	Small working group and plenum session
90 min	Dealing with resistance and disruptive factors, theme-centred interaction (TCI) [49]	Theoretical input, working on case studies in small working groups
30 min	Summary, outlook, evaluation	Plenum

In 2020 we added an input on online learning at the beginning of the second day, replaced the role play on group phases and team roles with team-building games and did more outdoor exercises due to the pandemic situation

### Data collection and study design

Between October 2016 and October 2020, we asked all participants in the interprofessional tutor qualification program to complete a paper-based questionnaire at the end of the interprofessional course. The questionnaire is comprised of the following sections:

1. Socio-demographic data (age, gender, study program and study semester) was collected to describe the participants.
2. General attitudes toward interprofessional learning were assessed with the Interprofessional Learning scale from the German version of the University of the West of England Interprofessional Questionnaire (UWE-IP-D) [50, 51]. The UWE-IP-D is an established assessment tool used at the University of Heidelberg to evaluate interprofessional learning environments. This questionnaire was validated for the German language in 2017 and was already available at the beginning of our study [52]. The statements of the UWE-IP-D subscale for Interprofessional Learning consists of nine items from 1 (= strongly agree) to 5 (= strongly disagree) with overall scores ranging from 9 to 45. Scores from 9 to 22, 23 to 31, and 32 to 45 indicate positive, neutral and negative attitudes, respectively [51]. We deleted individual UWE-IP-D subscales, if one or more single values were missing.
3. The assessment of the interprofessional setting was conducted on a five-point Likert scale (rating statement: Learning with other professions was successful) and an open-ended question (request: What has contributed to the success/failure of interprofessional learning?).
4. Tutor competency acquisition was assessed with eight rating questions related to the previously established competencies (five-point Likert scale; 1 = strongly agree, 5 = strongly disagree).
5. The teaching approach was assessed with nine rating questions on course design, interaction, satisfaction and overall rating of the training (five-point Likert scale; 1 = strongly agree, 5 = strongly disagree). To gain deeper insight into what contributed to the individual ratings, these two open-ended questions were added: “Which methods/content should definitely be retained?” and “How could the tutor training be improved?”.

### Data analyses

The qualitative data in the free-text fields were analysed using a content analytical approach. SZ and AH structured the text material regarding parts of the program that should be kept or improved. First, 88/86 statements for each question were screened and broken up into distinctive topics that the participants referred to in their feedback. Second, all statements were kept as worded and grouped thematically by both researchers independently. Each researcher identified the major themes or program components to which the statements referred and created main categories for each group of statements. Third, the two researchers compared and discussed their categorizations. To grasp the number of participants who expressed a specific feedback, all similar statements were counted and grouped into sub-categories. Both the quantitative description and the sub-categories were double-checked, discussed and refined in an iterative process [53].

We analyzed all quantitative data using IBM’s SPSS statistics (version 27, 2020) with a general significance level set to *α* = .05. A thorough data screening revealed no missing data, outliers, or nonlinear associations. Most Likert scale items showed floor effects. We computed relevant descriptive statistics and conducted inferential comparisons between medical students’ and healthcare students’ ratings. This mostly required non-parametrical statistics (Mann-Whitney tests and Kruskal-Wallis tests) only the UWE-IP-D scale allowed parametrical comparisons (students’ t-tests and ANOVAs). Since descriptive statistics for ordinal scales are often uninformative and the assumption of continuous latent variables seems plausible, we report means and standard deviations for the Likert scale items. To prevent inflation of false positive rates, we employed the Bonferroni-Holm adjustment for multiple comparisons [54] where necessary.

## Results

### Sample

A total of 77 students participated in the interprofessional tutor qualification program from October 2016 to October 2020. Seventy-five questionnaires were completed and included in this study (medical students, *n* = 75; HCS, *n* = 22; response rate 97%). The compositions of the cohorts and characteristics of the participants are shown in Table 2.

**Table 2:** Participants’ socio-demographic characteristics and cohort composition

	Medical students	HCS
Training participants	77	22
Per cohort		
2016	16	5
2017	15	4
2018	14	2
2019	10	9
2020	22	2
Questionnaires completed	75	22
Gender		
Number of responses ^a^	73	22
Male	34	2
Female	39	20
Age		
Number of responses ^a^	74	20
Mean	22.2	25.2
Range	19-31	21-38
Semester		
Number of responses ^a^	74	20
Mean	5.1	5.9
Range	3-12	3-9

*HCS* Healthcare students

^a^Varying sample size due to missing values

### General attitudes towards interprofessional learning

**Fig. 2:**
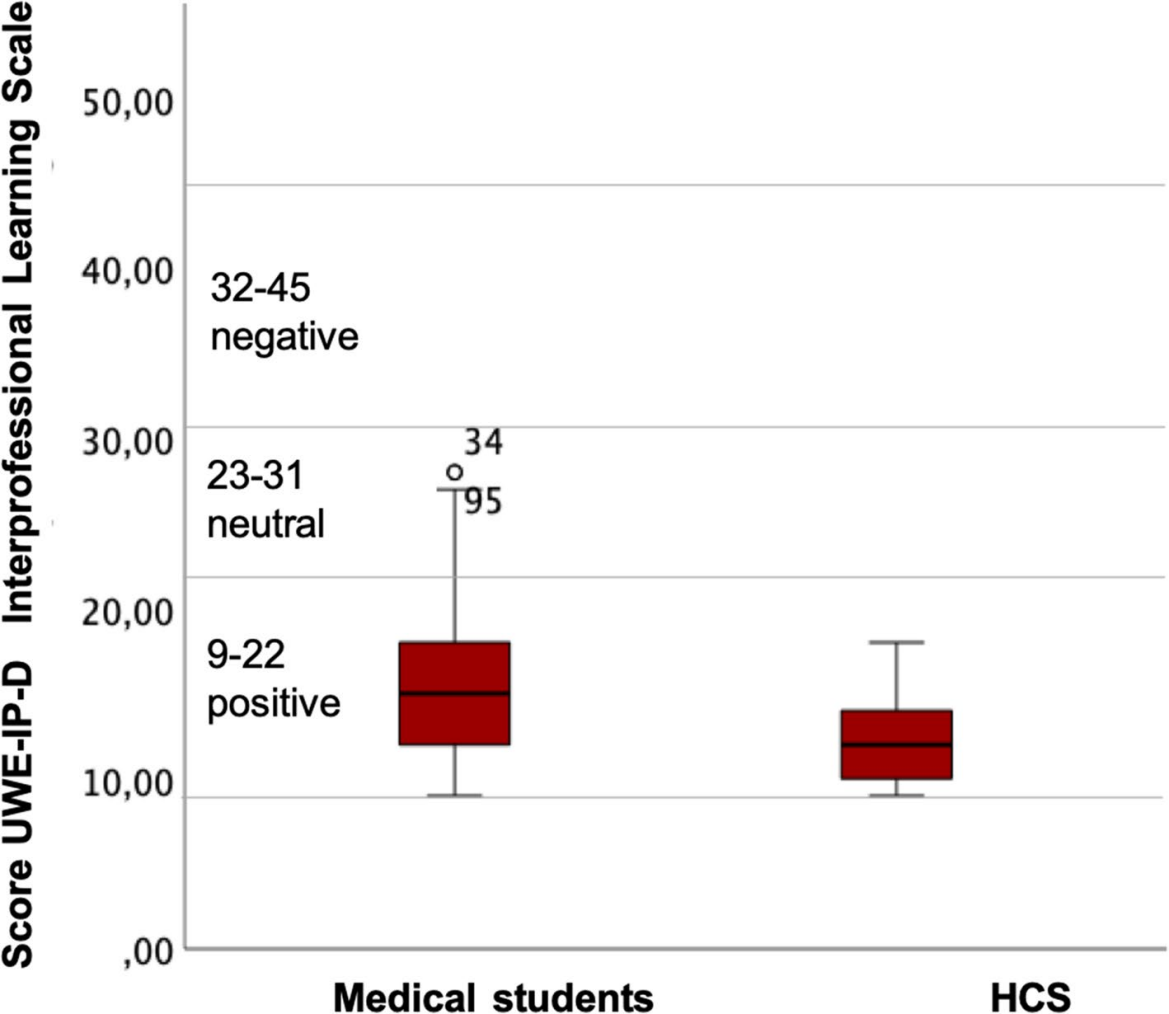
Attitudes toward interprofessional education. Medical students: *n* = 72; HCS (healthcare students): *n* = 21

Medical students reported higher values (*M* = 15.74) than HCS (*M* = 12.33) on the UWE-IP-D’s Interprofessional Learning Scale, *t*(60.4) = 4.01, *p* < .001, (*df*’s adjusted using Welch’s procedure for unequal variances). Low values indicate higher interest in Interprofessional Learning. Participants’ ratings did not differ across years, *F*(4, 89) = 1.036, *p* = .393, *n*.*s.* (Fig. 2).

### Assessment of the interprofessional setting

Overall, 66 students rated learning with other professions as successful (Likert scale 1 or 2), four students as partially successful (Likert scale 3), and no student as unsuccessful (Likert scale 4 or 5) (*M* = 1.33; *SD* = 0.56). The results of the quantitative assessment are shown in Table 3. In the free-text fields, 55 of the students with a positive rating gave reasons for their assessment, and all four students with a neutral rating.

**Table 3:** Assessment of the interprofessional setting

	1	Likert scale 2	3	n	mean	SD
Medical students	51 (71%)	17 (24%)	4 (6%)	72	1.35	0.46
HCS	15 (71%)	6 (29%)	-	21	1.29	0.46
Total	66 (71%)	23 (25%)	4 (4%)	93	1.33	0.56

Request: *I consider the learning with other professions as successful.* Five-point Likert scale (1 = strongly agree, 5 = strongly disagree)

*HCS* Healthcare students

1. The following reasons contributed most frequently to the positive assessment (at least two mentions):
2. Broadening horizons (31): get to know different experiences, exchange different points of view, gain new insights;
3. Participant characteristics (10): participants’ openness, nice people;
4. Trainer leadership behaviour (8): trainers are in charge of selecting the group composition, ensure a good group mix and respond to individual needs;
5. Group factors (7): group composition, mixing the professions within group work;
6. Group atmosphere (7): good group dynamics, excellent atmosphere.
7. Medical students with a neutral rating criticized the low number of students from the other professions (2) and found that the case studies did not always fit their own profession (2).

### Competency acquisition

Participants in the three study programs demonstrated good to very good competency acquisition in all areas. Data are shown in Table 4. No dependent variable showed significant differences between medical students and HCS after using Bonferroni-Holm’s adjustment for multiple comparisons [54].

**Table 4:** Competency acquisition

	Medical students			HCS			*U*	*P*
	n	mean	SD	n	mean	SD		
I know the characteristics of the tutor’s role.	75	1.20	0.43	22	1.18	0.39	819.0	.939
I am able to flesh out pre-structured tutorials.	75	1.75	0.66	22	1.95	0.95	753.5	.500
I am able to independently design tutorials entry and exit phases.	75	1,.4	0.55	22	1.23	0.53	645.5	.065
I am familiar with different mediation techniques and methods.	75	1.59	0.74	22	1.68	0.84	782.0	.680
I am able to recognize group dynamic processes.	75	1.51	0.62	22	1.41	0.50	777.0	.199
I am able to influence and control group dynamic processes.	74	2.00	0.64	21	2.24	0.70	651.5	.636
I am able to respond appropriately to participants’ different role behaviours.	75	1.89	0.67	22	2.05	0.65	735.5	.373
I identify with the tutor role.	75	1.24	0.49	22	1.50	0.74	685.0	.109

### Teaching approach

The teaching approach was consistently rated as very good. Here, too, no dependent variable showed significant differences between medical and other healthcare students after using Bonferroni-Holm’s adjustment for multiple comparisons [54]. Results are shown in Table 5.

**Table 5:** Assessment of design, interaction, teaching methods and students’ satisfaction

	Medical students			HCS			*U*	*P*
	n	mean	SD	n	mean	SD		
The overall concept of the training was clear.	75	1.37	0.56	22	1.09	0.29	622.0	.025
The trainers responded to participants’ questions and discussions.	75	1.04	0.20	22	1.09	0.43	819.0	.881
Fears and uncertainties were adequately addressed.	75	1.19	0.46	22	1.36	0.49	665.0	.050
Feedback was appropriate and constructive.	75	1.37	0.67	22	1.45	0.80	791.5	.716
The teamwork was based on partnership and goals.	75	1.15	0.36	22	1.09	0.29	779.0	.502
I was able to contribute my ideas during the learning process.	75	1.35	0.60	22	1.59	0.96	720.0	.268
The lecturers were good role-models for interprofessional cooperation.	75	1.16	0.44	22	1.32	0.65	742.5	.258
Attending the training was worthwhile.	75	1.41	0.70	22	1.50	0.86	803.5	.820
Learning with other professions was advantageous.	72	1.35	0.59	21	1.29	0.62	739.5	.848
Overall, I give the tutor training program the following grade^a^	74	1.31	0.49	21	1.33	0.58	776.0	.991

Five-point Likert scale (1 = strongly agree, 5 = strongly disagree)

*HCS* Healthcare students, *U* Test statistic for Mann-Whitney tests

^a^Five-point Likert scale (1 = very good, 5 = poor)

In an exploratory analysis we examined potential differences across cohorts on all dependent variables with Kruskal-Wallis tests. After adjusting for multiple comparisons [54], the only significant dependent variable was “Attending the tutor-training was worthwhile” *H*(4) = 18.795, *p* < .001; all other variables’ *H*’s ≤ 14.486, *n*.*s*. However, it did not show a consistent trend over time (*M*_2016_ = 1.16, *M*_2017_ = 1.90, *M*_2018_ = 1.31, *M*_2019_ = 1.74, *M*_2020_ = 1.13).

The open-ended question, “Which methods/content should definitely be retained?” was answered by 90 participants, and the question, “How could the tutor training be improved?” by 86. Results are shown in Table 6.

**Table 6:** Categorized content of free-text feedback

Categories	Feedback on what should remain part of the training	Feedback on what should be improved
**Didactics and teaching methods**		
Practical exercises and role plays in tutorials	*n* = 44	*n* = 18
	role plays evaluated positively (44)	more role play opportunities (8) clearer description of the task (7) additional topics to be thought of in the exercise tutorials (4) general dissatisfaction with role play (2)
Interactive games	*n* = 20	*n* = 5
	diverse icebreakers (e.g. sociometric, speed dating) (17) team-building games (6)^a^	include more methods (4) provide more activating methods to motivate participants (2)
Temporary division into small working groups	*n* = 12	*n* = 5
	small group work evaluated positively (12)	working group composition should be remixed more often during course time (3) shorten the sharing of experiences and working group results (2)
**Knowledge**		
Specific concepts and topics	*n* = 27	*n* = 3
	group dynamics and group roles (12) theme-centred interaction (Cohn) and analysis of challenging situations (11) basics of teaching and learning (6) leadership styles (6)	dive deeper into teaching and learning theory (3)
**Balance of the program**		
Balanced and entertaining	*n* = 21	*n* = 17
	methodological diversity and variety (13) interactive format (6) balanced relationship and connection between theory and practice (5)	provide less theoretical input (5) present theories in a more condensed and gripping way (4) provide more input (4) more exercises to practice the theories (3) too much content in general (2)
**Group and trainer**		
Interprofessionality and group characteristics	*n* = 11	*n* = 2
	positive atmosphere and pleasant interaction (6) group composition and interprofessionality (5)	some content should be more specific to my studies (2)
Trainer-trainee-interaction	*n* = 9	*n* = 10
	learner centred approach and individual feedback (7) support of multiple trainers (2)	feedback and reflection mentality too pronounced (10) criticized teaching styles of specific trainers (2)
**Program and organisation**		
Structure and design of the training	*n* = 5	*n* = 39
	clear structure (3) appropriate visual aids (3)	breaks management (15) time management (10) time of the training (afternoon blocks) not appropriate (5) shorten the training (4) less demanding content at the end (4) provide more written material (4) schedule and objectives should be provided in the beginning (2)
**Other**	everything was fine (6) consultation hour with experienced tutors (2) training for online teaching (2)^a^	more involvement of experienced tutors (2) more outdoor exercises (2)

*n* number of participants who referred to the category; in parentheses: number of mentions

^a^Part of the program since the 5th cohort; aspects mentioned only once are not included in the table

Many participants commented positively in the free-text fields on the role plays carried out. They could slip into the role of group leaders and participants and reflect on their experiences afterwards. Some wished for more detailed instructions for these role plays. Interactive games and methods were also well received. The majority of the inputs on theories and concepts, such as learning theory [42, 45, 55], theme-centred interaction [49], group dynamics [46], were also described as helpful. Many of the students praised the variety of methods presented to them during the training. Many students also liked the alternating work in small groups and plenum. Some participants would like to have more training content: more role plays, teaching methods, and theoretical input. However, there is also feedback that the amount of theory should be reduced. Some participants commented positively on the training group, mentioning that it was a pleasant atmosphere, and some explicitly said they appreciated its interprofessionality. Some perceived the pedagogical approach of the trainers as learner-centred, individual and supportive and therefore positive. Still, others said that the trainers’ feedback mentality was a bit too pronounced throughout the training. Many participants gave feedback on the training structure, notably that the breaks and time management could be improved (e.g., contradictory feedback regarding break length or frequency, timekeeping) and that the time of the training (in the afternoon) should be changed to the morning.

## Discussion

The overarching goal to redesign and implement an existing monoprofessional tutor qualification program to an interprofessional one was successfully achieved. The evaluation generally shows positive results and a high response rate, which points to the participants’ commitment and interest in the further development of teaching.

HCS scored slightly more positive (*M = 12.33*) on the Interprofessional Learning Scale (UWE-IP-D) than medical students (*M = 15.74*). This may be due to their familiarity with the theory and practice of interprofessionality. However, medical students are less likely to have the opportunity to learn together with other professions in a guided and systematic way. Overall, the attitudes of all training participants were significantly more positive than in most other studies on interprofessional learning [39, 56–58]. Comparably good values were only found regarding an interprofessional training ward [59] and a conversation training course for pre-licensure senior healthcare students [60]. Therefore, collaborative and active learning environments seem to foster interprofessional learning optimally.

Medical students and HCS appreciated the interprofessional setting and pointed out that their horizons were broadened by the exchange with students of other degree programs. This effect is also described in other studies [40, 61]. The willingness of students to engage in a nondiscriminatory exchange with each other even exceeded our expectations. It may have been advantageous that many of the students within the same study program were also unfamiliar before the course began. Also, we decided that participants would get to know each other (with the help of icebreaker exercises) without knowing about their respective memberships in different study programs.

Furthermore, the group composition was carefully designed to be as balanced as possible regarding gender and degree program in all small group exercises. Students mentioned that changing group compositions during the training challenged them to work together in different constellations. However, some students criticised the uneven design about the number of participants from other professions. Medical students, in particular, complained that a higher number of HCS would have facilitated interprofessional learning better.

In terms of teaching methods, students rated the group and role plays particularly positively. Interactive teaching methods have proven effective for interprofessional training in general [62], and role-plays be practical regarding communication skills [63, 64]. Many students particularly welcomed the mix of theory and practice and the exposure to specific concepts. By conveying new content to all participants, a common foundation was created on which the subsequent discussion and work processes were built. As a result, existing subject differences faded at times into the background. The program seemed to transcend subject boundaries: Students no longer seemed to be aware of their different degree programs while solving everyday tasks and slipping into the role of a future tutor. An awareness replaced the perception of differences that all future tutors have similar fears, goals and work processes to shape.

Khalili and Orchard stated that IPE helps to clarify one’s own professional identity and develop a dual identity. An “in-group” status substitutes out-group discrimination, and the professional identity is not lost but broadened, resulting in a “dual identity” [65, 66]. In this context, Khalili et al. stated that interprofessional interaction and critical reflection could cause an increased openness towards interprofessional collaboration. So-called transprofessional role-plays improve compassion and reliability across professions [67]. This study shows that a sense of belonging was developed between the students of the different study groups due to dealing with the new tutor role within role-plays and exchanges.

Most negative comments in free-text fields referred to “structure and organisation” despite our efforts to optimise the break times and the schedule. Evaluations of these topics seem very heterogeneous among the students, which may be due to different contextual conditions such as varying examinations schedules. No one way could be found to satisfy all students equally. However, it is not clear from this study whether the different perceptions regarding time management and feedback would have been equally evident in a monoprofessional setting. This survey also shows that contextual conditions, such as clear task descriptions, comprehensiveness of feedback and communicated time management, can be necessary for the positive evaluation of interprofessional training.

Overall, our study shows that the general attitude toward interprofessional learning and the reported learning achievements did not differ between tutors from different study programs. A multiprofessional trainer team, careful planning, balanced group composition and an interactive approach contribute substantially to the success of an interprofessional training program.

### Strengths and limitations

Almost all of the trained tutors in each of the five cohorts participated in the study allowing for a high degree of confidence in the gathered data. However, generalisation to other circumstances is impossible because we conducted the study at a single site. Furthermore, we did not complete a pre-post survey, and a control group was unavailable. Therefore, we cannot safely draw causal conclusions regarding the training’s effect, most importantly, whether didactic skills and positive attitudes toward interprofessional learning were already present before the qualification program. However, our qualitative data strongly suggest a significant positive impact on participants, in line with trainers’ impressions during the training. Another methodological limitation is that the results are based on self-assessments and statements gathered directly after the training. It would be interesting to analyse the long-term influence of interprofessional education on peer teaching and professional practice in a follow-up study.

In addition, we focused on whether opening up an existing program to other student groups was successful. In further studies, we would look more closely at precisely what shapes interprofessional attitudes, how mono-, inter- and transprofessional perceptions change over the course of the training, and what contributes to success.

## Conclusion

Overall, transforming an existing monoprofessional tutor qualification program into an interprofessional training program can foster tutor-specific and interprofessional competencies. The success of such training depends on balanced group compositions, contextual conditions and trainers’ leadership behaviour. Role-plays and other interactive teaching methods and opportunities to reflect on one’s future role were particularly suitable for promoting interprofessional exchange up to transprofessional exchange. The results provide important clues about the prerequisites and methodological considerations that could be important for the interprofessional redesign of existing training programs.

## Supplementary Information

**Additional file 1: Suplementary Table.** Evaluation Data from the cohorts 2016-2020.

## Data Availability

The dataset used and analysed during the current study is available as supplementary material.

## Abbreviations

AaL^Plus^: [Anatomie am Lebenden plus] Living Anatomy plus Anamnesis program
HCS: Healthcare students
IPE: Interprofessional education
UWE-IP-D: German version of the University of the West of England Interprofessional Questionnaire
